# Absolute vs Relative Income and Late-life Depressive Symptoms: Variations by Employment

**DOI:** 10.1093/geroni/igaf122.3042

**Published:** 2025-12-31

**Authors:** Veronica Howell, Benjamin Shaw

**Affiliations:** University of Illinois Chicago, Chicago, Illinois, United States; University of Illinois Chicago, Chicago, Illinois, United States

## Abstract

Prior research generally finds a positive association between income and well-being. This work builds on absolute and relative deprivation theories. By modeling absolute and relative income, this study examines whether these measures differ in association with late-life depressive symptoms and evaluates the role of employment. Data come from the National Social Life, Health, and Aging Project (NSHAP). Respondents reported baseline absolute income alongside income relative to other Americans and their broader social network. After controlling for covariates and baseline depressive symptoms, the association between income and later-life depressive symptoms was examined using linear regression. Five years after baseline, income relative to other Americans was significantly associated with increases in depressive symptoms (β = .432, p = .031), with no evidence of variation by baseline employment. Ten years after baseline, both income relative to other Americans (β = .760, p = .003) and relative to one’s own network (β =.598, p = .027) were significantly associated with increases in depressive symptoms. Interactions with employment were significant, indicating a reversal in the direction of these associations among those employed at baseline. These preliminary results suggest relative income may better predict depressive symptoms over time than absolute income. Further, both relative income measures suggest higher income is linked to reductions in depressive symptoms for those working during baseline. In comparison, higher income is associated with increased symptoms for those not working. Future research will explore how depressive and other mental health trajectories evolve based on income measures, employment transitions, and potential non-linear associations.

